# Facilitating translational science in anxiety disorders by adjusting extinction training in the laboratory to exposure-based therapy procedures

**DOI:** 10.1038/s41398-020-0786-x

**Published:** 2020-04-21

**Authors:** Maike Hollandt, Adrian Wroblewski, Yunbo Yang, Isabelle C. Ridderbusch, Tilo Kircher, Alfons O. Hamm, Benjamin Straube, Jan Richter

**Affiliations:** 1 Department of Biological and Clinical Psychology/Psychotherapy, University of Greifswald, Greifswald, Germany; 2 Department of Psychiatry and Psychotherapy, Philipps‐Universität Marburg, Marburg, Germany; 3 Center for Mind, Brain and Behavior (CMBB), Philipps‐Universität Marburg, Marburg, Germany

**Keywords:** Learning and memory, Human behaviour, Psychiatric disorders

## Abstract

Extinction learning is suggested to be a central mechanism during exposure-based cognitive behavioral psychotherapy. A positive association between the patients’ pretreatment extinction learning performance and treatment outcome would corroborate the hypothesis. Indeed, there is first correlational evidence between reduced extinction learning and therapy efficacy. However, the results of these association studies may be hampered by extinction-training protocols that do not match treatment procedures. Therefore, we developed an extinction-training protocol highly tailored to the procedure of exposure therapy and tested it in two samples of 46 subjects in total. By using instructed fear acquisition training, including a consolidation period overnight, we wanted to ensure that the conditioned fear response was well established prior to extinction training, which is the case in patients with anxiety disorders prior to treatment. Moreover, the extinction learning process was analyzed on multiple response levels, comprising unconditioned stimulus (US) expectancy ratings, autonomic responses, defensive brain stem reflexes, and neural activation using functional magnetic resonance imaging. Using this protocol, we found robust fear conditioning and slow-speed extinction learning. We also observed within-group heterogeneity in extinction learning, albeit a stable fear response at the beginning of the extinction training. Finally, we found discordance between different response systems, suggesting that multiple processes are involved in extinction learning. The paradigm presented here might help to ameliorate the association between extinction learning performance assessed in the laboratory and therapy outcomes and thus facilitate translational science in anxiety disorders.

## Introduction

The translation of neurobiological models of extinction learning to clinical applications has been emphasized as highly purposeful for improving the treatment of anxiety disorders^1,2^ but has not met expectations^3^. One reason might be the methodological gap between experimental protocols and treatment procedures. It is well accepted that extinction learning might be one central mechanism involved during exposure therapy^4,5^. Following this hypothesis, it can be hypothesized that individual differences in extinction learning performance prior to treatment could be associated with the outcome of exposure-based cognitive behavioral therapy (CBT). Therefore, treatment outcome prediction studies can make an important contribution in translational research. Indeed, there is evidence that deficits in extinction learning assessed in the laboratory prior to exposure therapy were related to poorer outcomes in some measures after exposure-based therapy in children with anxiety disorders^6^, adult individuals with elevated public-speaking anxiety^7^, individuals with spider fear^8^, and patients with panic disorder, and agoraphobia^9,10^. The problem with these correlational studies, however, is that they do not provide a better understanding of the behavioral, physiological, and neural mechanisms of extinction learning that might then help to improve extinction-like protocols in the clinic. The current study was designed to develop and test an extinction protocol that closely models the procedure used during exposure therapy and thus facilitates the translation of laboratory findings to clinical protocols.

During extinction training in the laboratory, the cue (conditioned stimulus) that has previously been paired with an unconditioned stimulus (US)—in the case of fear conditioning, the US is often a moderately painful stimulus—is now presented in the absence of the US. This prompts a complex-learning process, comprising several underlying (sub)processes^11^, during which a new extinction memory trace (CS+ predicts no_US) is formed, which then actively inhibits the excitatory memory trace (CS+ predicts US) that was established during fear acquisition training. Importantly, the methodological boundary conditions that are present during extinction training strongly contribute to the extinction learning processes^11,12^. This means that the extinction learning processes can vary as a function of the experimental conditions during the extinction training. In conclusion, harmonizing the experimental methodology between fear extinction paradigms would increase comparability, as recently highlighted for cross-species comparisons in translational science^13^. However, clinical exposure exercises predetermine specific framework conditions. To facilitate translation from experimental findings on mechanisms of extinction learning observed in the laboratory to exposure therapy, the correspondence between the boundary conditions of extinction training and exposure exercises should be optimized. Following this perspective, we developed and tested an extinction training procedure that modeled the exposure-based treatment procedures as accurately as possible.

First, exposure therapy initiates extinction learning in the context of long-lasting and well-consolidated fear memories. In contrast, most experimental studies employ extinction training immediately after fear acquisition, neglecting fear-memory consolidation. Importantly, basic research demonstrated that a delay between fear acquisition and extinction affects both time-course and end-point extinction performances as a function of the delay^12^.

Second, prior to exposure, patients are well aware of the stimuli they are afraid of, and fear responses are rather robust. In contrast, *noninstructed* fear acquisition training that is often used in conditioning studies results in large differences in learned fear responses between subjects, with some individuals not even showing a reliable fear response or having a declarative memory of the CS+/US contingency^14^. Anxiety patients show deficits in fear learning as indexed by less CS+/CS− discrimination due to deficits in inhibiting fear responses to the safety signal^15,16^. Extinction learning, however, can only be investigated in a meaningful way when fear responses are reliably acquired. Explicit instructions about the CS+/US contingency as implemented in instructed fear acquisition trainings facilitate conditioned fear acquisition^17^ and normalize dysfunctional responding in anxiety patients compared to unaffected controls^16^.

Third, during exposure therapy, patients are instructed prior to exposure to pay attention to feared stimuli and reflect on their central concerns. The explicit assessment of such central concerns prior to exposure of the fear cue might facilitate extinction learning and thus increase the efficacy of exposure therapy. In contrast, most extinction training protocols present both CS+ and CS− without any prior announcements. Thus, to facilitate translation to the clinical context, the experimental model should include a procedure where such central concerns are assessed prior to exposure. In some conditioning studies, such concerns are assessed by obtaining US expectancy ratings either isolated after a block of learning trials or concurrently on a trial-by-trial basis during the CS presentation. Whereas the block-based assessments only allow a rough estimate of changes in US expectancies, an assessment during the CS presentation provokes confounding between the processes of the risk assessment and those processes involved in activating behavioral, physiological, and neural patterns of the fear response. The current paradigm was designed to disentangle these processes and to model the clinical procedure as accurately as possible. A smaller sized CS was presented at the beginning of each trial, and the individual was asked to rate the probability that the US would follow such a stimulus. After this risk assessment, the CS was presented in its original size, and physiological response activation was measured on multiple response levels, including neural activation. With this procedure, we reduced possible confounding effects between those mechanisms that are involved in changing risk assessment and those that are active during extinction learning of physiological response activation. This is very important for better understanding the time course of changes in different components of the fear response often observed during exposure therapy.

Fourth, we included a return of fear test, a procedure assessing inhibitory memory recall^18^. This process is probably crucial for reducing the symptom relapse often observed after successful therapy^19^.

Fifth, patients’ excessive fear is expressed on multiple response levels. In line with these findings, previous studies included different measures of fear reduction as a result of extinction learning, including skin conductance responses (SCR)^6,8^, fear-potentiated startle (FPS)^8^, neural activity^7,9,10^, and subjective ratings of CS valence and arousal or US expectancy ratings^6–8^. However, different outcomes were demonstrated to be at least in part discordant^12^ and, thus, need to be assessed concordantly to better understand the mechanisms of change during exposure therapy.

Finally, recent studies differed in CS–US contingency rate during fear acquisition, which varied between 25%^7^ and 100%^6^. While low rates might hamper robust fear conditioning even during instructed acquisition, high rates facilitate subsequent extinction learning^12^ and delimitate the opportunity to map sufficiently subtle between-subject differences in the extinction learning curve due to ceiling effects. Therefore, a medium reinforcement rate and in addition, a sufficient number of extinction trials would be optimal.

Based on these principles, we developed an extinction-training procedure mapping the protocol more closely to the procedure of exposure therapy. To ensure that the fear response was reliably acquired prior to extinction learning, we explicitly instructed the participants which of the two stimuli was followed by the US and which was not. We also used a delayed extinction training procedure (extinction training started 24 h after the instructed acquisition) to ensure the consolidation of the fear memory. Furthermore, we started the extinction training after reactivating the fear memory because patients’ fear memories are always activated prior to exposure sessions by instructing patients about the upcoming exercises. Moreover, we assessed probability estimates of the US on a trial-by-trial basis prompted by a smaller image of the upcoming CS. This prior *risk assessment* procedure models the cognitive interventions assessing a patient's central concerns prior to and during exposure. By using this procedure, we disentangled risk assessment and activation of the physiological and neural components of the fear response to the cue.

The reduction in the fear response as a result of extinction learning was not only assessed on physiological and behavioral levels, but we also measured neural network activation patterns by scanning participants with 3 T functional magnetic resonance imaging (fMRI) during extinction training.

## Methods and materials

In two samples, we tested autonomic and behavioral indices of fear (study 1) and brain activity (study 2) using the new extinction training procedure. In the first study, 30 healthy students from the University of Greifswald (22 women; mean age 23.97 years; SD = 9.09) were allocated to one of two groups (immediate reinstatement: *N* = 14; delayed reinstatement: *N* = 16). For the assessment of brain activation, 16 healthy students (13 women; mean age 23.4 years, SD = 2.0 years) at the University of Marburg were included. The chosen sample sizes were based on previous studies that demonstrated robust effects of conditioned fear acquisition and extinction^20,21^. All participants gave written informed consent prior to the study. Local ethics committees at both sites approved the study protocols.

**Fig. 1:**
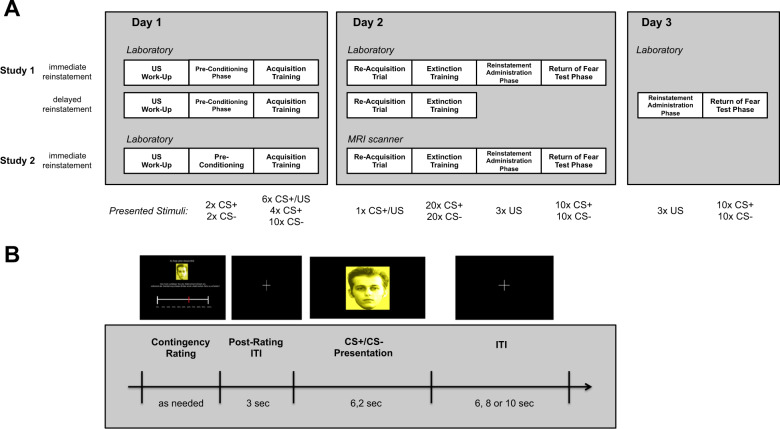
Experimental procedure of the extinction-training protocol. Panel **a**: Schematic illustration of the different phases in the optimized paradigm in the different experimental groups and the type and numbers of presented cues (CS+, CS−, US). Panel **b**: Schematic illustration of a single trial during fear acquisition in study 1. A prompting slide announced the next cue and was accompanied by a request of rating the expected US expectancy. After a following post-rating interval, the next CS was presented and in case of the CS+, accompanied by a US presentation in 60% of the presentations; the CS was followed by an intertrial interval. Startle probes were presented during 8 of 10 presentations of the CS+ and CS−, respectively, and 16 times during the ITIs. CS conditioned stimulus; US unconditioned stimulus; ITI intertrial interval.

The general procedure was virtually identical between studies. Figure 1a illustrates the phases of the experimental protocol, including the number of trials and type of stimuli presented in the different study groups. On day 1, all participants started with a US work-up procedure, during which the intensity of the US was adjusted individually to a level that was unpleasant but not painful^16^. After shock work-up, a preconditioning phase followed, during which CS+ and CS− were each presented twice without the US. Then, instructed acquisition training started. Here, participants were informed that the US would be presented at the end of CS+, but no information was given about how often the CS+ was paired with the US. Then, CS+ and CS− were presented 10 times each, and the US followed the CS+ during 6 of the 10 trials. On day 2, shock electrodes were attached to the same sites, and the experiment started with a single reacquisition trial, during which the CS+ was followed by the US, set to the intensity of the previous day. After the re-acquisition trial, extinction training started immediately without further instructions. CS+ and CS− were presented 20 times each without any US. After extinction training, a reinstatement administration phase followed during which the US was presented three times without any CS. In the following return of fear test phase, CS+ and CS− were again presented 10 times without US. Both phases were conducted immediately after the extinction training on day 2 (immediate reinstatement group in study 1 and study 2) or on a third assessment day (delayed reinstatement group in study 1; see Fig. 1a).

Figure 1b illustrates the structure of each specific trial starting with the presentation of a smaller version of the upcoming CS. Here, the participants were asked to rate the probability that this CS would be followed by the US when it would appear on the screen in full size (ratings did not involve any time restrictions). After a 3 sec post-rating period during which a fixation cross was presented, the CS was presented in full size for 6.2 sec. In study 1, the US expectancy ratings preceded every single CS presentation and were conducted on a visual analog scale (0–100%). In study 2, ratings on day 2 were conducted six times only in the MRI environment on a 10%-stepped scale: before and after reacquisition, after the 10th and 20th trials of extinction training, respectively, before and after reinstatement, and at the end of the experiment.

Two background-colored pictures of male faces with a neutral expression (from the Psychological Image Collection at Stirling; http://pics.stir.ac.uk, following ref. ^16^) served as CSs (counterbalanced between subjects; allocation followed an order specified before the study). CSs were presented for 6.2 and 6 sec in studies 1 and 2, respectively, followed by an intertrial interval (ITI, white fixation cross presented on a black screen) of 6–10 sec.

In study 1, a 50 ms burst of white noise with an intensity of 95 dB[A] (rise/fall < 1 ms) served as a startle-eliciting probe stimulus and was presented binaurally over Sennheiser AKG K66 headphones either 4.5 or 5 sec after CS onset and during the ITI (2, 3, 4, 5, or 6 sec after CS offset; *M* = 3.75; SD = 1.01; see supplements for more information).

The individually adjusted US (see supplements) was an electric shock train with a duration of 625 ms (125 single pulses) in study 1 and 500 ms (100 single pulses) in study 2, generated by a commercial stimulator (study 1: S48K; Grass Instruments, West Warwick, RI; study 2: DSA7, Digitimer, Medical Products, Wiesbaden) and applied to the forearm using a bar electrode (E.SB010, Digitimer, Letchworth Garden City, UK) and MRI compatible reusable cup electrodes (10 mm silver, Medical Products, Wiesbaden), respectively. The interstimulus interval between onset of the CS+ and the US was 5.6 and 5.5 sec in studies 1 and 2, respectively.

The physiological data in study 1 (eyeblink component of the startle reflex and the skin conductance response) and study 2 (blood-oxygen-level-dependent (BOLD) response) were recorded, preprocessed, and scored as described in the supplements. Data were analyzed using repeated-measures ANOVAs with Stimulus (CS+ vs. CS−, and—in the case of startle—vs. ITI) and Block (two trials per block except for the single reacquisition trial) as within-subjects factors. If indicated, analyses were followed by post hoc contrast analyses for the factor Block to test for systematic changes of stimulus (conditioning)— effects over blocks. To test for the effects of the reinstatement administration, mixed-model ANOVAs with Stimulus and Block (last extinction training trial vs. first trial of return of fear test phase) as the within-subjects factor and Time (immediate (day 2) vs. delayed reinstatement (day 3)) as the between-subjects factor were conducted.

To ensure that the amount of extinction learning was not confounded by differences in the amount of acquired fear (i.e., could be explained by the regression to the mean), we correlated US expectancy ratings obtained for the CS+ at the end of extinction training (last block of extinction training in study 1 and post extinction in study 2) with US expectancy ratings to CS+ (a) at the end of acquisition and (b) at the beginning (first block) of extinction (if extinction learning would be independent of the initial values, these correlations should be low and not significant). We also correlated the expectancy ratings of the last CS+ with the difference (delta change) scores between US expectancy ratings from initial to last block of extinction in study 1 and from pre-extinction to postextinction assessment in study 2. If changes in ratings during extinction portray the extinction learning process, these correlations should be significant. We limited these analyses to US expectancy ratings because of their relevance for the clinical data obtained during exposure exercises.

All tests were conducted two-sided and uncorrected for multiple comparisons. Prior to the analyses, the data were checked for potential violations of normal distribution and outliers based on Q–Q plots and box plots, respectively. All responses were in the expected range with no gross violations of normal distribution. A Greenhouse–Geisser procedure was used in case of a violation of the sphericity assumption in ANOVAs. Partial *η*_p_^2^ values are provided as a measure of effect size.

Regarding the MRI data, we defined specific contrasts: (1) Main effect ‘Stimulus Type’, comparing differences between CS+ and CS− in the different extinction training blocks, resulting in three *F*-tests and six post hoc *t*-tests; (2) Main effect ‘Time’, comparing two extinction blocks (early vs. late extinction including first and second half of extinction, respectively), resulting in three *F*-tests; (3) Changes during extinction training, resulting in three interactions (Time × Stimulus type) and four post hoc *t*-tests per interaction. All contrasts were assessed two-sided at *p* < 0.001 uncorrected and a cluster threshold of *k* = 20. Finally, we correlated BOLD activation during late extinction training with both activation during early extinction training and change in activation from early to late extinction training.

## Results

### Preconditioning phase and fear acquisition training (day 1)

**Fig. 2:**
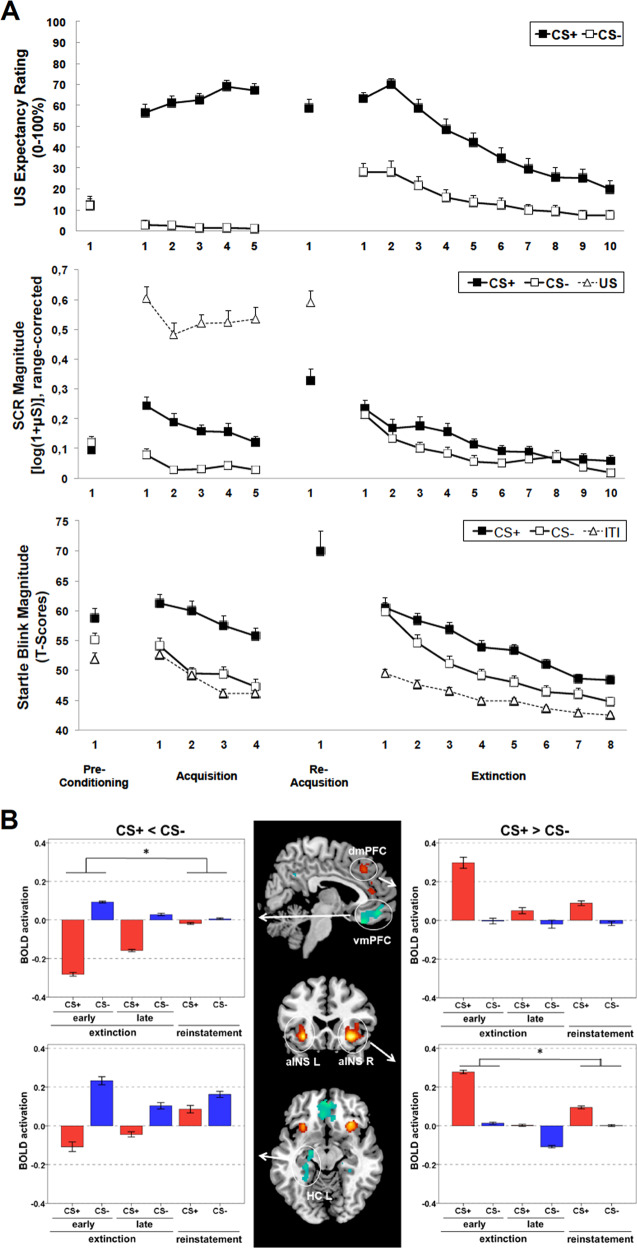
Subjective, physiological and neural responses during the extinction-training protocol. Panel **a**: Mean scores and standard errors for US expectancy ratings, SCRs and startle blink magnitudes, respectively, in study 1 as a function of stimulus type (CS+ and CS−, as well as US and ITI in case of SCR and startle, respectively) with two trials per block except for the single reacquisition trial. During phases of preconditioning, acquisition training, and extinction training, each block included two startle probes for both CS+ and CS− and four startle probes during the ITI. Panel **b**: BOLD group activation during early extinction training. BOLD activation assessed by the *t*-contrast CS + <CS− (*p* < 0.001 uncorr., *k* = 20) is illustrated in blue/green, and BOLD activation assessed by the *t*-contrast CS + > CS− (*p* < 0.001 uncorr., *k* = 20) is illustrated in red/yellow. The bar plots show mean extracted beta values and standard errors in early and late extinction training and during return of fear test procedure (*reinstatement*). During early extinction training, the ventromedial prefrontal cortex (vmPFC) and left hippocampus (HC L) were significantly more strongly activated for CS− than for CS+ (left), whereas the dorsomedial prefrontal cortex (dmPFC) and anterior insula (aINS) were significantly more strongly activated for CS+ than for CS−. Asterisks mark significant interactions (Stimulus×Time) between early extinction training and return of fear test phase (*p* < 0.001). The VmPFC was significantly more strongly activated for CS− than CS+ in early extinction, which diminished after reinstatement administration. Activity in the right aINS decreased for both CSs after reinstatement administration compared to early extinction with a stronger decrease to the CS+. CS conditioned stimulus; US unconditioned stimulus; ITI intertrial interval; SCR skin conductance response.

A detailed summary of the results is given in the supplement. Figure 2a illustrates the means for all dependent variables during CS+ and CS− for blocks of trials averaged across two trials in study 1. Mean blink magnitudes to startle probes presented during the ITIs are presented for startle data, and SCR to the aversive US are additionally presented in this figure. As expected, we observed robust acquisition of fear as indicated by increased US expectancy ratings for CS+ and decreased ratings for CS− from preconditioning to fear acquisition in both studies (study 1: Time × Stimulus *F*(1,29) = 172.90, *p* < 0.001, *η*^2^ = 0.86; study 2: Time × Stimulus *F*(1,15) = 12.40, *p* = 0.003, *η*^2^ = 0.45). In addition, strong acquisition effects were observed in study 1 for autonomic measures with significantly larger SCR to CS+ relative to CS− during acquisition (*F*(1,29) = 54.76, *p* < 0.001, *η*^2^ = 0.65). Moreover, there was a significant potentiation of the startle response evoked during CS+ relative to CS− (*F*(1,29) = 37.82, *p* < 0.001, *η*^2^ = 0.57). These data suggest that a robust fear-conditioned response to the CS+ was established.

### Reacquisition trial (day 2)

During the first and only reacquisition trial on day 2, we found a strong conditioned fear response to the CS+ in all indicators of the fear response (see middle section of Fig. 2; see supplements for details—we did not present the CS− during the reacquisition trial on day 2).

### Extinction training (day 2)

#### US expectancy ratings

During the following extinction training in study 1, the US expectancy ratings slightly increased during the first two blocks (*F*(1,29) = 3.73, *p* = 0.06, *η*^2^ = 0.11) for CS+ and then continuously decreased (linear trend: *p* < 0.001) but were still larger relative to CS− even after *20* extinction trials (*F*(1,29) = 8.89, *p* = 0.006, *η*^2^ = 0.24). These results were also supported by the ratings obtained in the MRI environment (study 2; see supplements and supplementary Fig. S1). The rated expectancies that the last CS+ would be followed by the US were not related to the US expectancies after acquisition (*r* = −0.15, *p* = 0.44) or at the beginning of extinction training (*r* = −0.01, *p* = 0.98). However, these expectancy ratings for the last CS+ at the end of extinction training were significantly negatively correlated with the amount of decrease (delta change scores) during extinction training (*r* = −0.81, *p* < 0.001), supporting the view that poorer extinction learning was not related to poorer acquisition. Expectancy ratings of the US following the CS+ varied between 0% and 99.5% for different subjects in the final extinction block, indicating high inter-individual variability (see Supplementary Fig. S2). These results were supported in study 2. Again, US expectancies during postextinction were not predicted by expectancy ratings prior to extinction (*r* = 0.30, *p* = 0.25) but were correlated with the decrease from preassessment to postassessment (*r* = −0.62, *p* < 0.05).

#### Physiological responses

In line with US expectancy ratings, FPS during the CS+ (relative to the ITI) continuously decreased during extinction training (linear trend: *p* < 0.001, *η*^2^ = 0.44) but was still significantly increased during the final extinction block (*F*(1,29) = 63.10, *p* < 0.000, *η*^2^ = 0.69). Interestingly, blink magnitudes and SCRs evoked during viewing the CS+ did not differ from response magnitudes evoked during CS− at the beginning of extinction training. This effect was due to an increase in responding not only to CS+ but also to CS−, suggesting a sensitization of physiological responses to both CS+ and CS− at the beginning of extinction training. Differential responses increased during intermediate extinction training (blocks 3–5; startle: *F*(1,29) = 19.43, *p* < 0.000, *η*^2^ = 0.40; SCR: *F*(1,29) = 8.12, *p* < 0.01, *η*^2^ = 0.22) and decreased again during the final blocks (no significant differences between CS+ and CS−).

#### Neural activation

Figure 2b illustrates the main BOLD activation results for early extinction training and activation changes over time. A detailed overview of all activation clusters and contrasts is given in Supplementary Tables S1–S3. During early extinction training, the *t*-contrast CS+ > CS− revealed significant activation in the bilateral anterior insula (aINS), rostral anterior cingulate cortex (rACC), and dorsomedial prefrontal cortex (dmPFC), whereas the ventromedial PFC (vmPFC) and left hippocampus (HC) showed significantly reduced activation for CS+ relative to CS− (significant contrast CS+ < CS−). Further activation was found in the posterior cingulate cortex and orbitofrontal cortex. During late extinction training, no significant activation clusters above the threshold were found for CS+ > CS−. However, several areas (see Supplementary Table S1), including the bilateral HC, showed significant activation for CS + < CS−. When investigating temporal effects between early and late extinction training (main effect of Time), we found a significant decrease in activation towards both CSs in the occipital cortex, bilateral aINS, dorsal, and rostral ACC, fusiform gyrus, inferior frontal gyrus, and left HC. The interaction (Stimulus × Time) between early and late extinction training only revealed significant activation in the left inferior frontal gyrus. Additional significant correlations demonstrated that low CS+-related brain activation during late extinction training was predicted by larger decreases in activation from early to late extinction training in the dmPFC (*r* = −0.60, *p* < 0.05), vmPFC (*r* = −0.91, *p* < 0.001), and left HC (*r* = −0.79, *p* < 0.001) but not by early extinction training activations (dmPFC: *r* = −0.05, *p* = 0.85; vmPFC: *r* = −0.36, *p* = 0.17; left HC: *r* = 0.06, *p* = 0.83), supporting the subjective data.

### Return of fear test phase (day 2 and day 3)

After the reinstatement administration phase, there was a return of the conditioned fear response indicated by an increase in US expectancy ratings to the CS+ relative to the CS− and a generalized increase in SCRs to both CS+ and CS− with no differences between reinstatement during day 2 or 3. Additionally, we found a general increase in FPS in the delayed reinstatement group irrespective of whether the probes were delivered during CS+ or CS−, suggesting an overall sensitization effect (see Supplemental material and Supplementary Fig. S3 for details of psychophysiological results and associated brain activation patterns).

## Discussion

In two virtually identical experimental settings, we demonstrated the feasibility of a new extinction-learning paradigm that was developed to increase the association between extinction learning processes engaged in the laboratory with those that are activated during exposure-based CBT. For this aim, those boundary conditions were considered during fear extinction training that are also present during clinical exposure exercises. Because boundary conditions strongly contribute to the extinction learning processes^11,12^, harmonizing the conditions between both the laboratory extinction training protocol and clinical exposure procedures should increase the association between experimental findings and clinical outcomes, and promoting the translation between basic science and clinical application is favored.

We observed a robust conditioned response during both the end of acquisition training and the recall of the fear memory after a consolidation period of 24 h in all dependent variables. After successful fear-memory recall, our fear extinction training resulted in an overall continuous decrease in physiological, behavioral, and cognitive indices of the conditioned fear response. Interestingly, we found slightly enhanced US expectancy ratings for the CS+ even after 20 extinction trials in ~60% of the subjects. Importantly, individual US expectancy ratings during late extinction training were independent of those during fear-memory recall but strongly correlated with the amount of decrease during extinction training, demonstrating that the paradigm was capable of differentiating extinction performances between subjects, independent of initial fear acquisition performance. Moreover, startle potentiation during CS+ was also still present at the end of the extinction period, suggesting that the extinction of subcortical defensive response activation is a rather slow-acting learning process (in dubio pro defensio^22^). The proposed protocol not only allows us to assess inhibitory fear learning on cognitive, behavioral, and autonomic fear response components but can also relate these indices of extinction learning to changes in brain activation.

During the first half of extinction training, we found stronger brain activation in the bilateral anterior insula, anterior cingulate cortex, and dorsomedial prefrontal cortex during CS+ compared to CS− processing. Previous studies have already observed activation patterns during fear extinction training (see ref. ^23^ for a recent meta-analysis). As highlighted by the meta-analysis, comparable brain activation during CS+ relative to CS− processing was, however, also observed during fear acquisition training in previous studies. Among other possible explanations, the authors speculated that persistent brain activity during fear extinction training reflects enduring but reduced threat processing to the CS+, suggesting fear memory recall. Similarly, we did not find comparable brain activation patterns during the second half of extinction training, suggesting that extinction learning indeed inhibited fear memory activation.

During the first half of extinction training, we also found decreased BOLD activity to the CS+ relative to the CS− in the ventromedial prefrontal cortex, supporting previous findings^21,24^. However, no meta-analytic evidence for a role of the vmPFC during extinction learning could be found^23^, suggesting effects of methodological specifications. The decreased activation of the vmPFC declined during extinction training. A parallel pattern of activation was found for the left hippocampus with decreased BOLD activity during CS+ relative to CS− at the beginning of extinction fading away towards the end, also supporting previous human^25–27^ and animal^28^ studies. In line with the US expectancy data, we also found that final CS+-related brain activation during late extinction training was predicted by the change in activation from early to late extinction training but not by early extinction training activation, which again demonstrates the independence of extinction-associated brain processes from initial fear memory recall in the paradigm used. Thus, the paradigm was demonstrated to be suitable to probe brain activation in the MRI environment that is specifically relevant during extinction learning and is independent from brain activation during fear acquisition and recall. Furthermore, a change in activation across extinction training phases was specifically detected in the left IFG, a region previously found to be affected by CBT during fear conditioning in patients with panic disorder and agoraphobia^29^.

During initial extinction training, autonomic and defensive reflex measures of fear did not discriminate between CS+ and CS−. This effect was driven by an increase in overall physiological response mobilization to the CS−, particularly illustrated by FPS to CS−, relative to the ITI, as also reported earlier^22,30^. Additionally, US expectancy ratings to the CS− strongly increased during initial extinction relative to the ratings obtained during fear acquisition training but still discriminated between cues. The subtle change in the learning context might explain this effect because—in contrast to the day before—no explicit instructions about the contingencies were given. Previous studies have demonstrated that explicit contingency reversal instructions are capable of reversing conditioned responses as measured by SCR^31–34^, startle reflex^35^, and verbal evaluations^36^. Although contingency reversal was not explicitly modeled, the lack of instruction might have resulted in an ambiguous state of US uncertainty followed by increased physiological responses to both CS+ and CS−. Importantly, comparable processes must be expected during exposure therapy, during which threat evaluations in patients are ambiguous regarding both danger and safety cues. Importantly, the observed discordance between different indices of the fear response suggests that the outcome measures might map different (sub)processes involved in fear extinction learning. The equal consideration of those different parameters in translational science might increase the understanding of specific mechanisms involved during extinction learning in the context of exposure therapy. At the same time, the observed discordance between response systems also highlights the need for a multilevel view of changes in fear responding during exposure exercises that is still almost exclusively based on reported symptoms and fear intensity.

The investigated sample was too small for extensive analyses of individual differences. Nevertheless, some preliminary analyses (see also supplemental information for additional post hoc analyses of the moderating effect of trait anxiety on extinction learning performance and Supplementary Fig. S4) suggest that the procedure used seems to be promising for assessing individual differences in extinction learning curves of different components of the fear response that might then be used as predictors for outcome of exposure-based treatment responses. Given the limited sample size and uncorrected multiple testing in our study, future research needs to replicate our results. Here, a critical comparison of the current new protocol with previously used fear-conditioning protocols should be considered. Moreover, comparing patients with anxiety disorders with healthy individuals might be helpful for detecting learning parameters that might be specifically relevant for translation to clinical procedures.

In summary, the presented procedure might foster the transition between basic and clinical science and thus, will complement the previous but limited evidence for extinction learning deficits to be a predictor of impaired exposure therapy^6–10^, as already started in a Germany-wide research consortium^37^.

## Supplementary information

Supplemental material

Table S1

Table S2

Table S3

Figure S1

Figure S2

Figure S3

Figure S4
