# Supplementary material for: Facilitating translational science in anxiety disorders by adjusting extinction training in the laboratory to exposure-based therapy procedures
Source: Transl Psychiatry. 2020 Apr 21;10:110. doi: 10.1038/s41398-020-0786-x (PMC7174283; doi:10.1038/s41398-020-0786-x)
Supplement: Supplementary file 1 — Supplemental material [file 41398_2020_786_MOESM1_ESM.doc]

**Facilitating translational science in anxiety disorders by adjusting extinction training in the laboratory to exposure-based therapy procedures**

Maike Hollandt, Adrian Wroblewski, Yunbo Yang, Isabelle Ridderbusch, Tilo Kircher, Alfons O. Hamm, Benjamin Straube, Jan Richter

**Supplemental Material**

**Study 1 (Psychophysiological assessment)**

**Method**

Participants

Thirty-seven healthy students (27 women) of the University of Greifswald reporting no current or past neurological/cardiovascular disease and psychotherapeutic treatment were recruited and participated for course credit or expense allowance. We excluded six subjects from the analysis due to missing data caused by the incorrect implementation of the study protocol and experimental equipment failures. We further excluded one subject from analysis who failed to participate on the second day of the experiment resulting in a final sample of 30 participants (22 women) between the age of 18 and 61 years (*M*=23.97; *SD*=9.09). Participants were randomized to one of two experimental groups that differed in the timing of the reinstatement test, either applied immediately (N=14) or 24 h (N=16) after extinction training. Groups did not significantly differ according to age and sex distribution. All participants signed an informed consent form prior to the study, which was approved by the Ethics Committee of the University of Greifswald, and were informed that they could withdraw from participation at any time.

Procedure

Upon arriving at the laboratory on day one, the participants first read and signed the informed consent form. Then, the electrodes for physiological sensors were attached. After the placement of a shock electrode to the left wrist, the US intensity was calibrated using a standardized shock work-up procedure ^1, 2^ to find the individual intensity that was rated as "highly aversive but not painful". During pre-conditioning phase, six startle probes were presented alone to ensure a stable baseline of blink magnitudes. Then CS+ and the CS- were presented twice with one startle probe presented during each CS and four probes delivered during the ITI. Participants were explicitly instructed that no electrical stimulation would occur during this phase. Afterwards participants were instructed that in the following acquisition phase shocks are possible only during the presentation of the CS+ (participants were informed about the contingency but not the reinforcement rate). During that phase, CSs were presented in a pseudo-randomized order ten times each with CS+ followed by the US in 60% of the trials. Startle probes were presented during 8 CSs trials each and 16 probes were presented during the ITI.

On day two (24h later), the sensors for physiological recording and the stimulation electrode were attached first. The position of the shock electrode was identical to the previous day as was US intensity. After presenting six startle probes alone, one initial CS+/US re-acquisition trial was presented also including a startle probe prior US presentation. Afterwards CS+ and CS- were presented 20 times each without any presentation of the US (uninstructed extinction phase). Participants were instructed that US presentation might be possible again, but no information about contingencies was given. Startle probes were presented during 16 of the CS+ and CS- trials, respectively, and 32 probes were delivered during ITI.

In those participants allocated to the immediate reinstatement group, the extinction training was followed by six startle probes to parallel the procedure in the delayed reinstatement group. Then three unpaired US presentation were presented repetitively during the ITI (reinstatement administration phase) and were followed by ten presentations of both the CS+ and CS- (return of fear test phase). Again startle probes were presented during eight of the ten CS+ and CS- trials, respectively, and 16 startle probes were delivered during the ITI. The delayed reinstatement group finished day 2 without the phases including the reinstatement administration and the return of fear test but was invited for day 3. On day 3, six startle probes were presented to ensure a stable baseline, followed by the reinstatement procedure described above.

During the whole experiment, participants were instructed to rate the probability for the occurrence of the US during CS presentation before each presentation of the CS+ and the CS-, respectively. For that, the presentation of the next CS was announced using a smaller picture of the respective CS accompanied by the sentence presented on the screen: “Now this picture is following.” The expectancy of the occurrence of the aversive US during the following CS was then rated on a visual analogue scale (0 – 100%) below the prompting CS picture using a computer mouse to move a cursor to the estimated percentage. The CS prompting slide was presented dynamically as long as needed for the rating and was followed by a fixed 3 s ITI before the (large) CS was presented.

Stimulus Materials

Two pictures of male faces with neutral expression (taken from the Psychological Image Collection at Stirling; [http://pics.stir.ac.uk](http://pics.stir.ac.uk/)) ^3^ and a colored background served as CSs. Assignment of stimuli to condition (CS+ vs. CS-) was counterbalanced between participants. The CSs were presented for 6200 ms, followed by an inter-trial interval (ITI, white fixation cross presented on a black screen) of 6 to 10 s (*M*=8.19; *SD*=1.71).

A 50 ms burst of white noise with an intensity of 95 dB[A] (rise/fall < 1ms) served as a startle probe and was presented binaurally over Sennheiser AKG K66 headphones either 4.5 s or 5 s after CS onset and during ITI (2, 3, 4, 5 or 6 s after CS offset; *M*=3.75; *SD*=1.01).

The US was a 625 ms electric shock train, composed of 125 single pulses of 2 ms and intervals of 3 ms between the pulses generated by a commercial stimulator (S48K; Grass Instruments, West Warwick, RI) and applied to the participants left forearm using a bar electrode (E.SB010, Digitimer, Letchworth Garden City, UK). During CS presentation US onset was 5600 ms after CS onset. The mean physical intensity of the US was 3.12 mA (SD=1.43).

Physiological Recordings and Apparatus

The eyeblink component of the startle reflex was measured by recording electromyographic (EMG) activity over the orbicularis oculi muscle beneath the left eye, using two Ag/AgCl surface electrodes (4mm diameter, F-E9-60, Warwick, RI), filled with electrolyte paste (GE Medical Systems Milwaukee, WI). The EMG raw signal was amplified and filtered through a 30 Hz to 1000 Hz bandpass filter using a Coulbourn S75-01 bioamplifier (Allentown, PA) and a 400 Hz Kemo-VFB-8-03 low pass filter (Kemo, Dartford, UK). The digital sampling with a rate of 1000 Hz started 100ms before and lasted until 100ms after the onset of the acoustic startle probe. The EMG signal was filtered offline using a 60 Hz highpass filter and was rectified and integrated (time constant: 10ms) by a digital filter.

To record skin conductance from the hypothenar muscle of the palmar surface of the participants' non-dominant hand ^4^, a Coulbourn S71-22 skin conductance coupler was used providing a constant 0.5 V across two Ag/AgCl surface electrodes (8 mm diameter, E224A, Warwick, RI; filled with isotonic 0.5 M sodium chloride electrode gel). The sampling rate was 10 Hz .

Data reduction and Response Definition

*Startle blink magnitudes*. Data were semi-automatically scored offline by using an in-house algorithm ^5^, that identified latency of blink onsets and peak amplitudes. The time window for startle response was defined between 20-120ms after startle probe onset and the magnitude had to peak within 150 ms after onset. No detectable eyeblinks were scored as zero responses (3.27% of all 4650 trials). Trials with excessive baseline activity, recording artifacts (e.g., electrode malfunctions) and spontaneous eyeblinks outside the latency window were treated as missings and therefore rejected (3.87%). All participants met the 80% criterion for valid responses and could be included in statistical analysis. The missing values were replaced individually for each subject by the overall mean blink response magnitude of this subject over all 155 trials of the experiment. Each response of each participant was then standardized and converted to T-Scores [50+(*z ×* 10)] to control for possible confounding effects of high inter-individual differences in baseline amplitude.

*Skin conductance response.* The skin conductance responses were scored as the first response in conductance within a 0.90 - 4.00 s time window following stimulus onset for CS and US, respectively, using an in-house program ^5^. Trials in which no SCR could be detected or with a response magnitude ≤ 0.04 µS were scored as zero responses (51.96% out of 2850 trials). There were no missing values for SCR over the entire experiment. Logarithms for each values were then computed to normalize the distribution ^6^. To reduce interindividual variability of the SCR that was not related to the conditioning and extinction tasks of the experiment, the log values were range corrected (division of individual score by the participants' maximum response within all CS and US trials) ^7^.

Physiological and rating data were reduced by averaging two trials (or in case of blink magnitudes two probes) to one block except during the one re-aquisition trial during fear memory recall.

Additional Analysis

In a post-hoc analysis, we tested the potential translational value of the paradigm by comparing participants reporting higher vs. lower trait anxiety. Moderating effects of trait anxiety - measured by the respective subscale of the State-Trait-Anxiety Scale ^8^ - on the course of extinction learning were tested with a repeated-measures ANOVA with Block (averaged across two trials) as a within-subject factor and group (high vs. low trait anxiety based on a median spit of STAI scores) as between-subject factor. In addition, correlation analyses between US expectancy ratings of each block and STAI scores were carried out. Based on the previous evidence^9^, we tested one-sided.

**Results**

Fear acquisition training (day 1)

*US expectancy ratings*. Despite explicit instructions about the absence of the US during pre-conditioning phase the US expectancy regarding both CSs was rated to be significantly larger than zero (CS+: *t*(29)=3.48, *p*=.002; CS-: *t*(29)=3.57, *p*=.001) but did not differ between CS+ and CS- (*F*(1,29)=0.233, *p*=.63, *η^2^*=.01). US expectancy ratings changed significantly during the course of acquisition training (Time x Stimulus *F*(1,29)=172.90, *p*<.001, *η^2^*=.86). While ratings of US expectancy significantly increased for CS+ (*F*(4,116)=5.33, *p*<.01, *η^2^*=.16) they remained on a stable low level for CS- (Time x Stimulus *F*(4,116)=6.82, *p*=.001, *η^2^*=.19).

*Skin conductance response.* SCRs to CS+ and CS- did not differ significantly during pre-conditioning. During the initial acquisition training, the US elicited strong SCRs that were reduced in the second block (*F*(1,29)=15.20, p=.001, *η^2^*=.34) but then remained unchanged between blocks 2 and 5, suggesting stable unconditioned responses across trials after initial sensitization. We found a significant increase in SCR magnitudes from pre-conditioning phase to initial fear acquisition training to the CS+ (*F*(1,29)=27.82, *p*<.001, *η^2^*=.49) but not to the CS- (overall Time x Stimulus *F*(1,29)=19.62, *p*<.001, *η^2^*=.40). Moreover, CS+ elicited significantly larger SCR magnitudes than CS- revealing a robust conditioning effect in this dependent variable (*F*(1,29)=54.76, *p*<.001, *η^2^*=.65).

*Startle blink magnitudes.* During startle habituation, blink magnitudes significantly decreased (*F*(2,58)=6.5, *p*<.01, *η^2^*=.18) to a stable baseline. During pre-conditioning phase the startle blink magnitudes were significantly potentiated during the presentation of the CS+ (*F*(1,29)=15.66, *p*<.000, *η^2^*=.35) and the CS- (*F*(1,29)=6.15, *p*<.05, *η^2^*=.18) as compared to ITI (overall: *F*(2,58)=9.11, *p*=.000, *η^2^*=.24). During acquisition training there was a reliable potentiation of the startle reflexes elicited during CS+ relative to both the CS- and the ITI (*F*(2,58)=47.56, *p*<.001, *η^2^*=.62), with no significant differences between CS- and ITI.

Extinction training (day 2)

*US expectancy ratings.* The rated US expectancy to the first CS+ (which was re-paired with the US during the presentation) was significantly lower than during the last fear acquisition training block (*F*(1,29)=5.37, *p*<.05, *η^2^*=.16) but did not differ to the first fear acquisition block. During the following initial extinction training block (including the first two unreinforced CS+ presentations) we found significantly higher US expectancy ratings for the CS+ than to the CS- (*F*(1,29)=46.86, *p*<.001, *η^2^*=.62). Although US expectancy to the CS- was significantly increased relative to the end of fear acquisition training (*F*(1,29)=39.68, *p*<.001, *η^2^*=.58) during the previous day. Between blocks 2 and 10 US expectancy continuously decreased for CSs with a greater decrease for CS+ relative to CS- resulting in a continuously decrease in discrimination (Stimulus x Time linear trend: *p*<.001, *η^2^*=.49). However, even after 20 extinction trials rated US expectancy was still significantly higher for the CS+ than for the CS- (*F*(1,29)=8.89, *p*<.01, *η^2^*=.24).

After excluding a non-extinguishing subject (US expectancy rating during the final extinction training block 10 was still about 99.5%; see figure S1) the course of CS+ related expectancy decrease was modulated by trait anxiety, high anxious subjects (N=15; m(STAI)=41.87, SD=6.69) showed a slower decline of US expectancy ratings relative to low anxious subjects (N=14; m(STAI)=32.00, SD=3.11) although both groups showed comparable expectations towards the end of extinction training (quadratic trend: p=.03; see figure S4). These results were confirmed by correlational analyses: trait anxiety was negatively correlated with the rated US expectancies during intermediate extinction training (block 5: r=.33; p=.043; block 6: r=.33, p=.039; block 7: r=.33, p=.043). No significant correlations were obtained during early and late extinction, respectively.

*Skin Conductance Response.* During the initial re-acquisition trial, mean SCR to the US was as high as during the initial acquisition training block. Moreover, SCR magnitudes to the initial CS+ were significantly larger than SCRs to the CS+ during the last block (*F*(1,29)=17.92, *p*<.001, *η^2^*=.38) and the first block of fear acquisition training (*F*(1,29)=4.96, *p*<.05, *η^2^*=.15). SCR significantly decreased from initial CS+ presentation to the CS+ during the first extinction training block (*F*(1,29)=4.93, *p*<.05, *η^2^*=.15). During the first and second block the SCRs did not differ between CS+ and CS- but did significantly discriminate during block 3 (*F*(1,29)=5.41, *p*<.05, *η^2^*=.16), block 4 (*F*(1,29)=5.80, *p*<.05, *η^2^*=.17) and block 5 (*F*(1,29)=6.32, *p*<.05, *η^2^*=.18) with stronger responses to CS+ relative to CS-. During the following blocks 6 to 10, no SCR discrimination was observed, again.

*Startle blink magnitudes.* During startle habituation, the blink magnitudes again decreased (*F*(2,58)=19.25, *p*<.001, *η^2^*=.40) to a stable baseline. There was a significant potentiation of blink magnitudes during the initial and re-paired CS+ relative to both the first (*F*(1,29)=5.29, *p*<.05, *η^2^*=.15) and the last (*F*(1,29)=12.82, *p*=.001, *η^2^*=.31) fear acquisition training block. Startle response magnitudes elicited during the CS+ significantly decreased from re-acquisition trial to the first extinction training block (*F*(1,29)=6.10, *p*<.05, *η^2^*=.17). Startle blink magnitudes did not differ between CS+ and CS- during the first extinction block but were strongly potentiated to both CS+ and CS- relative to ITI (*F*(2,58)=32.12, *p*<.001, *η^2^*=.53). During the course of extinction training startle discrimination between the CS+ and CS- increased until block 3 and then further decreased between blocks 5 and 8 resulting in significant quadratic trend (Stimulus x Time: *p*<.01, *η^2^*=.24). Fear potentiation of the blink magnitudes evoked during CS+ and CS- relative to the ITI continuously decreased during extinction training (linear Stimulus x Time trend: CS+: *p*<.001, *η^2^*=.44; CS-: *p*<.001, *η^2^*=.45). During the last extinction training block, startle blink magnitude was still significantly potentiated during the CS+ as compared to both CS- (*F*(1,29)=12.77, *p*=.001, *η^2^*=.31) and ITI (*F*(1,29)=63.10, *p*<.000, *η^2^*=.69). Also, startle response during the CS- was still higher than during the ITI (*F*(1,29)=5.85, *p*<.05, *η^2^*=.17).

Reinstatement test (day 2 or day 3)

*US expectancy ratings*. The reinstatement administration phase was followed by an increase in US expectancy ratings to both CS+ and CS- which was more pronounced for the CS+ (Stimulus x Time *F*(1,29)=3.62, *p*=.067, *η^2^*=.11; see supplemental figure S3). No significant differences between reinstatement groups were observed.

*Skin Conductance Response.* The CS related SCRs significantly increased after reinstatement administration (*F*(1,28)=11.06, *p*<.01, *η^2^*=.28; figure S3) with no significant differences between CS+ and CS- and reinstatement group.

*Startle blink magnitudes.* Figure S3 illustrates mean scores and standard errors of the startle blink magnitudes during the last test trial prior to and the first trial after the reinstatement administration phase elicited during CS+, CS-, and the ITI, respectively, as a function of reinstatement group. In addition, the blink magnitudes elicited during the last two startle alone presentations during the startle habituation phases before the reinstatement administration phase were presented as a reference.

Due to habituation effects on the startle the blink magnitudes during the startle habituation phase prior reinstatement administration were significantly lower in the immediate reinstatement group (conducted after several startle probe presentations during extinction training on day 2) as compared to the delayed reinstatement group (conducted at the beginning on day 3 without any other startle probe presentations; *F*(1,28)=18.94, *p*<.001, *η^2^*=.40). As compared to the two last startle probes during startle habituation phase the blink magnitudes were substantially higher during the two ITI startle probes after the reinstatement administration phase (*F*(1,28)=22.05, *p*<.001, *η^2^*=.44) with no significant differences between reinstatement groups indicating a context sensitization following the three US alone presentations during the reinstatement administration phase.

If comparing the CSs related fear-potentiated startle (relative to the ITI) prior and after reinstatement administration phase we found significant group differences (Time x Group *F*(1,28)=4.03, *p*=.05, *η^2^*=.13): fear potentiation startle (relative to the ITI) decreased in the immediate reinstatement group but increased in the delayed reinstatement group (see figure S3). This effect was observed to both CS types.

**Study 2 (fMRI assessment)**

**Methods**

Participants

Sixteen students (13 women; 12 right-handed subjects; mean age 23.4 years, *SD*=2.0 years) of the University of Marburg with no histories of medical or mental illnesses were included. All subjects had normal or corrected to normal visual acuity and were naive to the experiment beforehand. Written informed consent was obtained prior to the participation in the study, and the study protocol was approved by the local ethics committee according to the declaration of Helsinki.

Procedure

The experimental procedure was adopted from study 1 to the MRI environment with subtle methodological adaptations in a) timing of stimulus presentation, b) mode of contingency ratings during extinction, c) absence of acoustic probe stimuli at day 2, and d) settings for the electric stimuli. As in study 1, the paradigm consisted of two major phases: fear acquisition training on the first day in an experimental room outside the MRI scanner and fear extinction training immediately followed by the reinstatement procedure 24 hours later in the MRI environment.

The same stimuli serving as CSs were used as in study 1. In study 2, each stimulus was presented for 6 s with an inter-stimulus interval (ITI) of 6-10 s. During these intervals, subjects were instructed to fixate a superimposed white cross on a black background. An unpleasant electricocutaneous stimulus was used as US with a stimulus duration of 500 ms, composed of 100 single pulses of 5 ms. It was presented after 5.5 s after stimulus onset so that it co-terminated with the CS+. For the application, a constant current stimulator (DS7A, Digitimer, Medical Products, Wiesbaden) with MRI compatible reusable cup electrodes (10 mm silver, Medical Products, Wiesbaden) and specially produced electrode gel were used. The electric stimuli were triggered by the presentation software and delivered to the forearm of the non-dominant hand. The current intensity was adjusted according to the procedure described for study 1.

Experimental procedure on the first day was the same as in study 1. Extinction training on day 2 took place in the MRI scanner 24 hours after the fear acquisition training. Instructions (uninstructed extinction training) for the subjects and experimental procedure were comparable to those in study 1. Using the Presentation 17.2 software (Neurobehavioral Systems, Albany, CA, http://www.neurobs.com/) all stimuli inside the MR scanner were presented on an MRI-compatible LCD screen (LG SL9000, 60 Hz, 4:3, 1024 x 786 pixels), visible through a mirror attached to the MR head coil. Initially, one CS+ follwed by the US was presented (re-acquisition). Next, three experimental phases followed: two extinction training phases (early vs. late) with 10 CS+ and 10 CS- presentations each without any CS-US coupling, a reinstatement administration phase comprising presentation of the three electric stimuli during ITI (black screen), and again followed by a return of fear test phase with 10 CS+ and 10 CS- without the US. In study 2, we did not include a second experimental group to investigate reinstatement on the third day. The current intensity and electrode position were adopted from the adjustments of the first day. Contingency ratings about US expectancy took place at six time points during day 2: Prior to and after re-aquisition, between early and late extinction training, prior to and after reinstatement administration phase, and at the end of the experiment. Due to technical limitations inside the scanner an 11-point scale had to be used, allowing ratings in steps of 10%. Furthermore, the subjects had only 5 s for the rating to shorten the length of the fMRI paradigm. No startle probes were presented on the second day.

MRI data acquisition

As the focus of Study 2 was on fear extinction, MRI data were only acquired and analyzed on the second day. Time courses of subjects’ brain activity were acquired using a 3-Tesla MR scanner (Siemens Magnetom TIM Trio, Erlangen) equipped with a 12 channel head matrix receive coil at the Department of Psychiatry and Psychotherapy, University of Marburg. Functional images were obtained using a T_2_*-weighted gradient-echo echo-planar imaging (EPI) sequence sensitive for the BOLD contrast (TE = 30 ms, TR = 2000 ms, flip angle 90°, matrix size 64 x 64 voxels, voxel size 3.6 x 3.6 x 4.0 mm, slice thickness 4.0 mm, inter-slice gap 0.4 mm, field of view (FOV) = 230 mm, 33 slices, ascending phase encoding direction). Slices were positioned transaxially parallel to the intercommissural (AC-PC) plane and tilted 20° to reduce magnetic susceptibility artifacts in prefrontal areas. In total, 455 volumes were collected. Four subjects were measured with a different MR sequence to test for the best adjustments. TR was changed to 1600 ms and TE to 25 ms leading to a total number volume number of 570. In addition, for each subject an additional high-resolution structural image was acquired using a three-dimensional T_1_-weighted magnetization-prepared rapid gradient-echo (3D MPRAGE) sequence in sagittal plane (TE = 2.26 ms, TR = 1900 ms, inversion time (TI) = 900 ms, flip angle 9°, matrix size 256 x 256 voxels, voxel size 1 x 1 x 1 mm, slice thickness 1.0 mm, FOV = 256 mm, 176 slices). Total scanning time for the extinction paradigm was 15 min and 4:30 min for the structural scan.

Data analysis

US expectancy ratings were analyzed using IBM SPSS Statistics (version 20). Analysis routines were the same as in Study 1. Functional MRI data were preprocessed and analyzed using the SPM12 software package (Statistical Parametric Mapping, Wellcome Trust Center for Neuroimaging, London, UK; http://www.fil.ion.ucl.ac.uk) based on Matlab R2016a (version 9.0.0; MathWorks). After discarding the first five volumes to minimize T1-saturation effects, all images were high-pass filtered (cut-off period 128 s), co-registered to the anatomical T1 images, segmented, spatially realigned and normalized into the MNI space using the MNI template (resulting voxel size 2x2x2 mm3). As this paradigm was developed for the use in a large multi-center study with seven different centers ^10^, spatial smoothing followed an iterative approach recommended for multi-center studies ^11^ with a target kernel of 8 mm isotropic Gaussian filter. Thereby, we wanted to achieve comparability with future analyses in multi-center studies. Furthermore, as an iterative smoothing procedure takes differences in intrinsic smoothness ^12^ of the data into account, it might reduce variance induced by smoothness even in the context of a single center study.

The statistical whole-brain analysis was performed in a two-level, mixed-effects procedure. The voxel-wise BOLD activity was modeled by means of a single subject first-level General Linear Model (GLM) comprising the onsets of the US, rating phases and CS+/CS- for each block respectively, resulting in 8 experimental regressors. The six realignment parameters were additionally included as nuisance covariates to account for residual motion artifacts. The hemodynamic response was modeled by the canonical hemodynamic response function (HRF). Parameter estimate (ß-) images for the HRF were calculated for each condition and each subject. Parameter estimates for the six relevant conditions (CS+ and CS- for each block) were then entered into a within-subject flexible factorial analysis. As there were no significant differences in brain activations between the two sub-groups (TR=2s vs. TR=1.6s), we analyzed these data sets together.

Additional Analysis

In a post-hoc analysis we correlated the trait anxiety score as measured by the State-Trait-Anxiety Scale with US expectancy ratings and brain activation. Again, we tested one-sided. Due to the small sample size, we here abstained from a between-group comparison.

**Results**

Fear acquisition training (day 1)

*US expectancy ratings.* Supplemental figure S2 shows mean scores and standard errors of US expectancy ratings during all phases including pre-conditioning and fear acquisition training on day 1, and pre and post the re-aquisition trial, and after early and late extinction and reinstatement, respectively, regarding both CS+ and CS-. In line with the results of study 1, although subjects were instructed about the absence of the US during pre-conditioning, shock expectancy ratings to both CSs were significantly above zero (CS+: *t*(15)=8.37, *p*<.001; CS-: *t*(15)=3.72, *p*<.01). US expectancy to the CS+ was rated significantly higher than to the CS- (*F*(1,15)=13.71, *p*<.01, *η^2^* = .48).

As compared to pre-conditioning, the CS differentiation in expectancy ratings significantly increased during the first fear acquisition training block (Time X Stimulus *F*(1,15)=12.40, *p*<.01, *η^2^*=.45) with increasing and decreasing expectancies to the CS+ and CS-, respectively. During the course of fear acquisition training the ratings further increased for the CS+ (*F*(4,60)=11.10, *p*<.001, *η^2^*=.43) and decreased for the CS- (*F*(4,60)=3.80, *p*<.01, *η^2^*=.20; overall: Time X Stimulus *F*(4,60)=12.74, *p*<.001, *η^2^*=.46). After the last acquisition training block, shock expectancy ratings to the CS- did not differ significantly from zero.

Extinction training (day 2)

*US expectancy ratings.* Immediately prior to the re-acquisition trial US expectancies to the CS+ and CS- were rated higher and lower, respectively, as compared to end of fear acquisition training (Stimulus X Time *F*(1,15)=5.04, *p*<.05, *η^2^*=.25). The US expectancy regarding the CS+ was still higher as to the CS- (*F*(1,15)=32.41, *p*<.001, *η^2^*=.68). As a function of re-acquisition the US expectancy for CS+ increased significantly (*F*(1,15)=4.62, *p*<.05, *η^2^*=.24), but remained stable for CS-.

Prior to the initial extinction training block, we found a significant difference between US expectancy ratings for CS+ and CS- (*F*(1,15)=58.48, *p*<.001, *η^2^*=.80). In the course of extinction training (after two training phases comprising 10 extinction trials each) expectancy ratings to both CSs decreased significantly (*F*(2,30)=67.72, *p*<.001, *η^2^*= .82) with a stronger decrease for CS+ (Time X Stimulus *F*(2,30)=14.68, *p*<.001, *η^2^*=.50). At the end of the second extinction phase ratings for CS- did not differ significantly from zero.

After the reinstatement administration phase, we observed a significant increase in US expectancy rating difference between CS+ and CS- (Time X Stimulus *F*(1,15)=5.95, *p*<.05, *η^2^*=.28). In more detail, CS+ ratings significantly increased (*F*(1,15)=6.43, *p*<.05, *η^2^*=.30) but CS- ratings remained stable. At the end of extinction training expectancy ratings to the CS- did not differ significantly from zero. However, US expectancy ratings were still higher for the CS+ relative to the CS- (*F*(1,15)=8.14, *p*<.05, *η^2^*=.35). Increasing STAI scores went along with increasing CS+ related US expectancy ratings prior to both the re-acquisition trial (r=.54, p<.05) and extinction training (r=.51, p<.05).

*fMRI results.* During early extinction training, we found activation in most relevant regions of the fear network. The t-contrast CS+ > CS- revealed significant activation in bilateral anterior insula (aINS), rostral anterior cingulate cortex (rACC) and dorsomedial prefrontal cortex (dmPFC), whereas ventromedial PFC (vmPFC) and left hippocampus (HC) showed significant activation for CS+ < CS-. Further relevant activation clusters were found in the posterior cingulate cortex and orbitofrontal cortex.

During late extinction training no significant activation clusters above the threshold were found for CS+ > CS-, but bilateral HC showed significant activation for CS+ < CS- (see supplementary table S1). When investigating temporal effects between early and late extinction training (main effect Time; see supplementary table S2), we found a general significant decrease of activation towards both CSs in the occipital cortex, bilateral aINS, dorsal and rostral ACC, fusiform gyrus, inferior frontal gyrus and left hippocampus (see supplementary table S3). The interaction (Stimulus X Time) between early and late extinction training only revealed significant activation in the left inferior frontal gyrus (table S3). Increasing STAI scores went along with increasing decrease of activations in the left insula from early to late extinction (r=.61, p<.01).

During the return of fear test phase, we observed significant differential activation between CS+ and CS- mainly in the inferior frontal gyrus and bilateral aINS, both resulting from the t-contrast CS+ > CS- (table S1). Activation changes between late extinction training and return of fear test phase were observed only in right lingual gyrus, right fusiform gyrus and occipital cortex (table S2). The interaction (Stimulus X Time) between late extinction training and return of fear test phase revealed no further relevant activation clusters (table S3). However, as compared to early extinction training, we found significant differential activation changes in vmPFC and right aINS (figure 2B). The differential effect in the vmPFC (strong deactivation to the CS+ and activation to the CS-) during early extinction training diminished completely during return of fear test phase. In the right aINS we observed activation decrease to both CSs during return of fear test phase as compared to early extinction training with a significantly stronger decrease to the CS+ (figure 2B).

**References**

1. Heitland I, Groenink L, Bijlsma EY, Oosting RS, Baas JM. Human fear acquisition deficits in relation to genetic variants of the corticotropin releasing hormone receptor 1 and the serotonin transporter. *PLoS One* 2013; **8**(5)**:** e63772.

2. Vervliet B, Kindt M, Vansteenwegen D, Hermans D. Fear generalization in humans: impact of verbal instructions. *Behaviour research and therapy* 2010; **48**(1)**:** 38-43.

3. Duits P et al. Enhancing Effects of Contingency Instructions on Fear Acquisition and Extinction in Anxiety Disorders. *Journal of Abnormal Psychology* 2017; **126**(4)**:** 378-391.

4. Boucsein W et al. Publication recommendations for electrodermal measurements. *Psychophysiology* 2012; **49**(8)**:** 1017-1034.

5. Globisch J, Hamm A, Schneider R, Vaitl D. A computer programm for scoring reflex eyeblink and electrodermal responses written in Pascal. *Psychophysiology* 1993; **39**(Suppl)**:** S30.

6. Venables PH, Christie MJ. Electrodermal activity. *Techniques in psychophysiology* 1980; **54**(3).

7. Lykken DT, Venables PH. Direct measurement of skin conductance: A proposal for standardization. *Psychophysiology* 1971; **8**(5)**:** 656-672.

8. Laux L, Glanzmann P, Schaffner P, Spielberger CD. *Das state-trait-angstinventar: STAI*. Beltz Weinheim, 1981.

9. Lonsdorf TB, Merz CJ. More than just noise: Inter-individual differences in fear acquisition, extinction and return of fear in humans - Biological, experiential, temperamental factors, and methodological pitfalls. *Neurosci Biobehav Rev* 2017; **80:** 703-728.

10. Heinig I et al. Optimizing exposure-based CBT for anxiety disorders via enhanced extinction: Design and methods of a multicentre randomized clinical trial. *International Journal of Methods in Psychiatric Research* 2017; **26**(2).

11. Friedman L, Glover GH, Krenz D, Magnotta V, First B. Reducing inter-scanner variability of activation in a multicenter fMRI study: role of smoothness equalization. *Neuroimage* 2006; **32**(4)**:** 1656-1668.

12. Friston KJ, Jezzard P, Turner R. Analysis of functional MRI time-series. *Human brain mapping* 1994; **1**(2)**:** 153-171.

**Table S1.** Peak MNI coordinates, cluster sizes, F-values, and *t*-values, respectively, for activated brain regions reflected in the comparison between stimuli. Cluster sizes are defined as the number of voxels in MNI space at a voxel size of 2 x 2 x 2 mm³. Ex1 = early extinction training, Ex2 = late extinction training, ROF = return of fear test phase.

**Table S2.** Peak MNI coordinates, cluster sizes, F-values, and *t*-values, respectively, for activated brain regions reflected in the comparison between extinction blocks. Cluster sizes are defined as the number of voxels in MNI space at a voxel size of 2 x 2 x 2 mm³. Ex1 = early extinction training, Ex2 = late extinction training, ROF = return of fear test phase.

**Table S3.** Peak MNI coordinates, cluster sizes, F-values, and *t*-values, respectively, for activated brain regions reflected in the interaction between time and stimulus type. Cluster sizes are defined as the number of voxels in MNI space at a voxel size of 2 x 2 x 2 mm³. Ex1 = early extinction, Ex2 = late extinction, ROF = return of fear test phase.

**Figure S1.** Mean scores and standard error of US shock expectancy ratings in study 2 as a function of stimulus type (CS+ and CS-) with two ratings per block during phases of pre-conditioning phase and acquisition training, and one rating per block during re-acquisition, extinction training, and return of fear test phase. CS, conditioned stimulus.

**Figure S2.** Frequency distribution of rated US probabilities for the CS+ (categorized into intervals of 10 %) by subjects during the last extinction training block. CS, conditioned stimulus; US, unconditioned stimulus.

**Figure S3.** Mean scores and standard errors for US expectancy ratings, SCRs, and startle blink magnitudes during the trials prior and after the reinstatement administration phase, respectively, as a function of stimulus type (CS+ and CS-, and ITI in the case of startle). For both US expectancy ratings and SCRs responses were summarized for both groups (immediate vs. delayed reinstatement) but separated in case of startle. CS, conditioned stimulus; US, unconditioned stimulus; ITI, inter-trial interval; SCR, skin conductance response.

**Figure S4.** Mean scores and standard errors for US expectancy ratings to the CS+ as a function of trait anxiety group (high vs. low) during extinction training with two trials per block. CS, conditioned stimulus; US, unconditioned stimulus.
