# Supplementary material for: Facilitating translational science in anxiety disorders by adjusting extinction training in the laboratory to exposure-based therapy procedures
Source: Transl Psychiatry. 2020 Apr 21;10:110. doi: 10.1038/s41398-020-0786-x (PMC7174283; doi:10.1038/s41398-020-0786-x)
Supplement: Supplementary file 2 — Table S1 [file 41398_2020_786_MOESM2_ESM.docx]

| anatomical regions/ | Peak MNI coordinates | | | Cluster size | F-values/ | p-value cluster |
| --- | --- | --- | --- | --- | --- | --- |
| hemisphere | x | y | z | (no. voxels) | *t*-values | FWE corrected |
| *Main Effect: Stimulus Type*  *Ex1: CS+ vs. CS-* |  |  |  |  | F-values | p -values |
| Anterior insula R | 32 | 26 | -6 | 436 | 43.38 | < .001 |
| Anterior insula L | -30 | 22 | -8 | 222 | 31.96 | .004 |
| Ventromedial prefrontal cortex L | -6 | 46 | -18 | 628 | 25.77 | < .001 |
| Hippocampus L | -20 | -12 | -16 | 55 | 23.43 | .421 |
| Supramarginal gyrus R | 58 | -46 | 32 | 26 | 21.70 | .865 |
| Posterior cingulate cortex L | -6 | -54 | 24 | 131 | 21.43 | .041 |
| Precentral gyrus L | -12 | -20 | 70 | 23 | 17.93 | .904 |
| Thalamus L | -22 | -34 | -2 | 143 | 17.46 | .029 |
| Postcentral gyrus R | 48 | -10 | 40 | 65 | 16.81 | .310 |
| Rostral anterior cingulate cortex R | 6 | 42 | 12 | 24 | 16.34 | .891 |
| Inferior temporal gyrus L | -38 | -60 | 0 | 33 | 16.28 | .757 |
| Dorsomedial prefrontal gyrus R | 4 | 30 | 40 | 43 | 15.93 | .594 |
| Posterior cingulate cortex R | 10 | -46 | 6 | 27 | 13.91 | .850 |
| *Post-hoc t-tests*  *Ex1: CS+ > CS-* |  |  |  |  | *t*-values |  |
| Anterior insula R | 32 | 26 | -6 | 592 | 6.59 | < .001 |
| Anterior insula L | -32 | 22 | -8 | 290 | 5.65 | .002 |
| Supramarginal gyrus R | 58 | -46 | 32 | 35 | 4.66 | .767 |
| Putamen R | 16 | 4 | -4 | 28 | 4.12 | .859 |
| Rostral anterior cingulate cortex R | 6 | 42 | 12 | 54 | 4.04 | .510 |
| Dorsomedial prefrontal cortex R | 4 | 30 | 40 | 67 | 3.99 | .369 |
| Middle temporal gyrus R | 56 | -28 | -6 | 26 | 3.78 | .882 |
| *Ex1: CS+ < CS-* |  |  |  |  |  |  |
| Ventromedial prefrontal cortex L | -6 | 46 | -18 | 738 | 5.08 | < .001 |
| Hippocampus L | -20 | -12 | -16 | 74 | 4.84 | .308 |
| Posterior cingulate cortex L | -6 | -54 | 24 | 209 | 4.63 | .011 |
| Precentral gyrus L | -12 | -20 | 76 | 47 | 4.23 | .601 |
| Thalamus L | -22 | -34 | -2 | 222 | 4.18 | .008 |
| Postcentral gyrus R | 48 | -10 | 40 | 90 | 4.10 | .202 |
| Inferior temporal gyrus L | -38 | -60 | 0 | 50 | 4.03 | .561 |
| Thalamus R | 18 | -30 | -6 | 70 | 3.87 | .341 |
| Orbitofrontal cortex R | 28 | 32 | -16 | 21 | 3.82 | .932 |
| Posterior cingulate cortex R | 10 | -46 | 6 | 47 | 3.73 | .601 |
| Precentral gyrus R | 42 | -14 | 60 | 20 | 3.72 | .941 |
| Angular gyrus L | -42 | -70 | 22 | 26 | 3.70 | .882 |
| Inferior frontal gyrus L | -50 | 42 | 4 | 21 | 3.48 | .932 |
| *Main Effect: Stimulus Type*  *Ex2: CS+ vs. CS-* |  |  |  |  | F-values |  |
| Middle temporal gyrus R | 50 | -56 | -2 | 64 | 29.78 | .320 |
| Precuneus R | 6 | -52 | 58 | 129 | 18.74 | .043 |
| Angular gyrus L | -38 | -66 | 24 | 96 | 17.51 | .117 |
| Precuneus L | -10 | -44 | 58 | 144 | 17.39 | .028 |
| Middle temporal gyrus L | -46 | -62 | 2 | 30 | 17.26 | .805 |
| Angular gyrus R | 44 | -60 | 20 | 61 | 16.97 | .351 |
| Superior frontal gyrus R | 22 | -2 | 64 | 29 | 16.85 | .821 |
| Hippocampus L | -26 | -42 | -8 | 29 | 16.34 | .821 |
| Hippocampus R | 30 | -48 | -4 | 27 | 15.81 | .850 |
| Superior parietal lobe R | 30 | -52 | 58 | 32 | 15.78 | .774 |
| Posterior cingulate cortex R | 12 | -50 | 6 | 21 | 14.45 | .926 |
| *Post-hoc t-tests*  *Ex2: CS+ > CS-* |  |  |  |  | *t*-values |  |
| No significant activation |  |  |  |  |  |  |
| *Ex2: CS+ < CS-* |  |  |  |  |  |  |
| Middle temporal gyrus R | 50 | -56 | -2 | 83 | 5.46 | .243 |
| Precuneus R | 6 | -52 | 58 | 306 | 4.33 | .002 |
| Angular gyrus L | -38 | -66 | 24 | 205 | 4.18 | .012 |
| Precuneus L | -10 | -44 | 58 | 254 | 4.17 | .004 |
| Middle temporal gyrus L | -46 | -62 | 2 | 44 | 4.15 | .642 |
| Angular gyrus R | 44 | -60 | 20 | 98 | 4.12 | .164 |
| Superior frontal gyrus R | 22 | -2 | 64 | 42 | 4.11 | .670 |
| Hippocampus L | -26 | -42 | -8 | 82 | 4.04 | .263 |
| Hippocampus R | 30 | -48 | -4 | 46 | 3.98 | .614 |
| Superior temporal gyrus R | 24 | 8 | 54 | 24 | 3.95 | .904 |
| Posterior cingulate cortex R | 12 | -50 | 6 | 45 | 3.80 | .628 |
| Superior frontal gyrus R | 24 | 8 | 54 | 24 | 3.69 | .904 |
| Occipital cortex R | 32 | -72 | 38 | 20 | 3.44 | .941 |
| Posterior cingulate cortex L | -12 | -52 | 4 | 21 | 3.34 | .932 |
| *Main Effect: Stimulus Type*  *ROF: CS+ vs. CS-* |  |  |  |  | F-values |  |
| Supramarginal gyrus L | -54 | -50 | 30 | 42 | 16.60 | .610 |
| *Post-hoc t-tests*  *ROF: CS+ > CS-* |  |  |  |  | *t*-values |  |
| Inferior frontal gyrus L | -52 | 36 | 0 | 20 | 4.08 | .941 |
| Supramarginal gyrus L | -54 | -50 | 30 | 63 | 4.07 | .409 |
| Superior temporal gyrus R | 64 | -30 | 4 | 35 | 4.03 | .767 |
| Anterior insula R | 34 | 22 | -14 | 27 | 3.62 | .871 |
| Anterior insula L | -28 | 22 | -8 | 20 | 3.50 | .941 |
| *ROF: CS+ < CS-* |  |  |  |  |  |  |
| No significant activation |  |  |  |  |  |  |
