# Supplementary material for: Facilitating translational science in anxiety disorders by adjusting extinction training in the laboratory to exposure-based therapy procedures
Source: Transl Psychiatry. 2020 Apr 21;10:110. doi: 10.1038/s41398-020-0786-x (PMC7174283; doi:10.1038/s41398-020-0786-x)
Supplement: Supplementary file 3 — Table S2 [file 41398_2020_786_MOESM3_ESM.docx]

| anatomical regions/ | Peak MNI coordinates | | | Cluster size |  | p-value cluster |
| --- | --- | --- | --- | --- | --- | --- |
| hemisphere | x | y | z | (no. voxels) | F-values | FWE corrected |
| *Main Effect: Time*  *Ex1 vs. Ex2* |  |  |  |  |  | p -values |
| Occipital cortex R | 22 | -94 | 6 | 405 | 33.70 | < .001 |
| Anterior insula R | 40 | 8 | -6 | 874 | 32.57 | < .001 |
| Fusiform gyrus R | 40 | -56 | -16 | 134 | 31.45 | .037 |
| Anterior insula L | -34 | 8 | -2 | 328 | 30.08 | < .001 |
| Occipital cortex L | -16 | -92 | 2 | 429 | 27.66 | < .001 |
| Dorsal anterior cingulate cortex R | 12 | 28 | 30 | 345 | 25.62 | < .001 |
| Lingual gyrus L | -10 | -70 | -16 | 206 | 25.66 | .005 |
| Superior temporal gyrus R | 48 | -26 | 0 | 176 | 23.54 | .011 |
| Hippocampus L | -20 | -32 | -8 | 118 | 20.99 | .060 |
| Rostral anterior cingulate cortex R | 4 | 38 | 6 | 22 | 20.99 | .915 |
| Fusiform gyrus L | -34 | -54 | -22 | 216 | 20.58 | .004 |
| Cerebellum R | 10 | -68 | -32 | 65 | 20.52 | .310 |
| Thalamus R | 6 | -14 | -2 | 154 | 20.42 | .021 |
| Calcarine cortex L | -8 | -72 | 12 | 70 | 17.91 | .265 |
| Cerebellum L | -14 | -68 | -32 | 23 | 15.97 | .904 |
| Precuneus R | 26 | -58 | 12 | 20 | 14.98 | .936 |
| *Ex 2 vs. ROF* |  |  |  |  |  |  |
| Lingual gyrus R | 4 | -60 | -6 | 26 | 19.72 | .865 |
| Fusiform gyrus R | 38 | -54 | -16 | 30 | 17.30 | .805 |
| Occipital cortex R | 26 | -98 | -10 | 33 | 16.29 | .757 |
| *Ex1 vs. ROF* |  |  |  |  |  |  |
| Thalamus R | 6 | -12 | -2 | 73 | 22.60 | .241 |
| Orbitofrontal cortex R | 32 | 48 | -12 | 47 | 21.34 | .532 |
| Anterior insula L | -34 | 8 | 0 | 35 | 17.44 | .725 |
| Anterior insula R | 36 | 2 | 0 | 49 | 16.86 | .503 |
| Postcentral gyrus R | 62 | -16 | 34 | 27 | 16.23 | .850 |
| Hippocampus L | -30 | -20 | -4 | 44 | 16.04 | .578 |
| Inferior frontal gyrus L | -48 | 10 | 4 | 21 | 15.77 | .926 |
