# Supplementary material for: Facilitating translational science in anxiety disorders by adjusting extinction training in the laboratory to exposure-based therapy procedures
Source: Transl Psychiatry. 2020 Apr 21;10:110. doi: 10.1038/s41398-020-0786-x (PMC7174283; doi:10.1038/s41398-020-0786-x)
Supplement: Supplementary file 4 — Table S3 [file 41398_2020_786_MOESM4_ESM.docx]

| anatomical regions/ | Peak MNI coordinates | | | Cluster size | F-values/ | p-value cluster |
| --- | --- | --- | --- | --- | --- | --- |
| hemisphere | x | y | z | (no. voxels) | *t*-values | FWE corrected |
| *Interaction: Time X Stimulus Type*  *Ex1 vs. Ex2 X CS+ vs. CS-* |  |  |  |  | F-values | p-values |
| Inferior frontal gyrus L | -44 | 40 | 0 | 112 | 22.58 | .071 |
| *Post-hoc t-tests*  *CS+: Ex1 > Ex2* |  |  |  |  | *t*-values |  |
| Occipital cortex R | 22 | -92 | 4 | 335 | 5.20 | < .001 |
| Superior temporal gyrus R | 48 | -26 | 0 | 305 | 5.20 | .002 |
| Cerebellum L | -8 | -72 | -18 | 767 | 5.12 | < .001 |
| Dorsal anterior cingulate cortex R | 12 | 28 | 30 | 449 | 5.03 | < .001 |
| Anterior insula R | 36 | 6 | -4 | 1252 | 5.00 | < .001 |
| Anterior insula L | -36 | 10 | -2 | 468 | 4.93 | < .001 |
| Rostral anterior cingulate cortex R | 4 | 38 | 6 | 49 | 4.80 | .574 |
| Fusiform gyrus R | 40 | -56 | -14 | 75 | 4.72 | .300 |
| Occipital cortex L | -14 | -92 | 4 | 319 | 4.62 | .001 |
| Calcarine cortex L | -6 | -72 | 12 | 165 | 4.33 | .030 |
| Calcarine cortex R | 10 | -68 | 18 | 250 | 4.33 | .005 |
| Dorsolateral prefrontal cortex L | -38 | 46 | 26 | 55 | 4.07 | .498 |
| Brain stem | -6 | -32 | -6 | 28 | 3.94 | .859 |
| Inferior frontal gyrus R | 52 | 18 | 14 | 22 | 3.92 | .923 |
| Hippocampus L | -24 | -26 | -4 | 29 | 3.72 | .846 |
| *CS-: Ex1 > Ex2* |  |  |  |  |  |  |
| Occipital cortex R | 22 | -94 | 6 | 347 | 5.35 | < .001 |
| Fusiform gyrus R | 40 | -56 | -16 | 242 | 5.31 | .005 |
| Anterior insula R | 40 | 8 | -6 | 640 | 5.19 | < .001 |
| Occipital cortex L | -28 | -78 | -10 | 405 | 5.00 | < .001 |
| Anterior insula L | -34 | 8 | -2 | 137 | 4.95 | .060 |
| Lingual gyrus L | -10 | -66 | -14 | 119 | 4.89 | .095 |
| Thalamus L | -20 | -32 | -6 | 179 | 4.75 | .022 |
| Fusiform gyrus L | -32 | -48 | -20 | 95 | 4.50 | .177 |
| Middle temporal gyrus L | -46 | -24 | -8 | 28 | 4.48 | .859 |
| Dorsal anterior cingulate cortex R | 10 | 4 | 32 | 142 | 4.45 | .053 |
| Inferior frontal gyrus R | 42 | 28 | 8 | 38 | 4.24 | .726 |
| Thalamus R | 14 | -34 | 0 | 24 | 4.16 | .904 |
| Superior temporal gyrus L | -54 | -42 | 8 | 27 | 3.94 | .871 |
| Inferior frontal gyrus L | -48 | 10 | 6 | 51 | 3.79 | .548 |
| Postcentral gyrus L | -52 | -14 | 44 | 62 | 3.77 | .419 |
| Superior temporal gyrus R | 44 | -22 | -6 | 26 | 3.75 | .882 |
| Precentral gyrus R | 44 | -10 | 30 | 41 | 3.62 | .684 |
| *CS+: Ex1 < Ex2* |  |  |  |  |  |  |
| No significant activation |  |  |  |  |  |  |
| *CS-: Ex1 < Ex2* |  |  |  |  |  |  |
| No significant activation |  |  |  |  |  |  |
| *Interaction: Time X Stimulus Type*  *Ex2 vs. ROF X CS+ vs. CS-* |  |  |  |  | F-values |  |
| Superior temporal gyrus R | 48 | -30 | 4 | 87 | 18.94 | .155 |
| Superior frontal gyrus R | 22 | -2 | 64 | 23 | 16.75 | .904 |
| Precuneus R | 8 | -50 | 56 | 46 | 16.60 | .547 |
| *Post-hoc t-tests*  *CS+: Ex2 > ROF* |  |  |  |  | *t*-values |  |
| No significant activation |  |  |  |  |  |  |
| *CS-: Ex2 > ROF* |  |  |  |  |  |  |
| Middle temporal gyrus L | -44 | -66 | 6 | 27 | 4.18 | .870 |
| *CS+: Ex2 < ROF* |  |  |  |  |  |  |
| Superior temporal gyrus R | 48 | -32 | 2 | 121 | 4.74 | .090 |
| Fusiform gyrus L | -36 | -44 | -22 | 42 | 4.57 | .670 |
| Postcentral gyrus L | -6 | -42 | 64 | 58 | 4.40 | .463 |
| Lingual gyrus L | -4 | -68 | -4 | 114 | 4.31 | .108 |
| Cerebellum L | -2 | -66 | -42 | 34 | 4.17 | .781 |
| Occipital cortex R | 24 | -96 | 10 | 66 | 4.14 | .379 |
| Superior parietal lobe R | 20 | -56 | 58 | 38 | 4.12 | .726 |
| Precentral gyrus R | 32 | -24 | 70 | 25 | 4.07 | .893 |
| Cuneus R | 16 | -70 | 18 | 85 | 4.03 | .230 |
| Fusiform gyrus R | 28 | -54 | -16 | 40 | 3.99 | .698 |
| Middle frontal gyrus L | -26 | 20 | 30 | 23 | 3.90 | .914 |
| Calcarine cortex L | -6 | -70 | 14 | 55 | 3.68 | .498 |
| *CS-: Ex2 < ROF* |  |  |  |  |  |  |
| Fusiform gyrus R | 38 | -54 | -16 | 21 | 3.50 | .932 |
| *Interaction: Time X Stimulus Type*  *Ex1 vs. ROF X CS+ vs. CS-* |  |  |  |  | F-values |  |
| Angular gyrus L | -38 | -56 | 34 | 76 | 21.10 | .219 |
| Inferior frontal gyrus L | -52 | 40 | 0 | 37 | 20.08 | .692 |
| Ventromedial prefrontal cortex L | -4 | 46 | -18 | 100 | 18.09 | .103 |
| Ventromedial prefrontal cortex R | 4 | 30 | -22 | 43 | 15.63 | .594 |
| Anterior insula R | 32 | 28 | -2 | 38 | 15.44 | .675 |
| *Post-hoc t-tests*  *CS+: Ex1 > ROF* |  |  |  |  | *t*-values |  |
| Thalamus R | 6 | -12 | -2 | 62 | 4.23 | .419 |
| Inferior frontal gyrus L | -48 | 10 | 2 | 35 | 3.99 | .767 |
| Anterior insula R | 30 | 14 | 4 | 23 | 3.88 | .914 |
| Anterior insula L | -34 | 8 | 0 | 35 | 3.85 | .767 |
| *CS-: Ex1 > ROF* |  |  |  |  |  |  |
| Putamen L | -28 | -20 | -2 | 120 | 4.47 | .092 |
| Thalamus R | 6 | -12 | -2 | 63 | 4.31 | .409 |
| Fusiform gyrus L | -30 | -78 | -6 | 51 | 4.02 | .548 |
| Precentral gyrus R | 40 | -8 | 36 | 55 | 4.01 | .498 |
| Putamen R | 30 | -14 | -4 | 60 | 3.99 | .441 |
| Anterior insula R | 38 | 2 | 0 | 101 | 3.97 | .151 |
| Thalamus L | -18 | -36 | -6 | 32 | 3.93 | .808 |
| Anterior insula L | -34 | 8 | 2 | 21 | 3.77 | .932 |
| Inferior frontal gyrus R | 52 | 38 | 8 | 20 | 3.70 | .941 |
| Cerebellum L | -14 | -66 | -16 | 21 | 3.67 | .932 |
| Superior temporal gyrus R | 50 | -24 | 0 | 20 | 3.66 | .750 |
| Superior temporal gyrus L | -60 | -44 | 10 | 25 | 3.52 | .750 |
| *CS+: Ex1 < ROF* |  |  |  |  |  |  |
| Orbitofrontal cortex R | 32 | 48 | -12 | 35 | 4.57 | .767 |
| Superior frontal gyrus L | -16 | 32 | 52 | 23 | 3.82 | .914 |
| *CS-: Ex1 < ROF* |  |  |  |  |  |  |
| Ventrolateral prefrontal cortex R | 40 | 46 | -2 | 67 | 3.94 | .369 |
