# Supplementary figures and images for: Facilitating translational science in anxiety disorders by adjusting extinction training in the laboratory to exposure-based therapy procedures
Source: Transl Psychiatry. 2020 Apr 21;10:110. doi: 10.1038/s41398-020-0786-x (PMC7174283; doi:10.1038/s41398-020-0786-x)

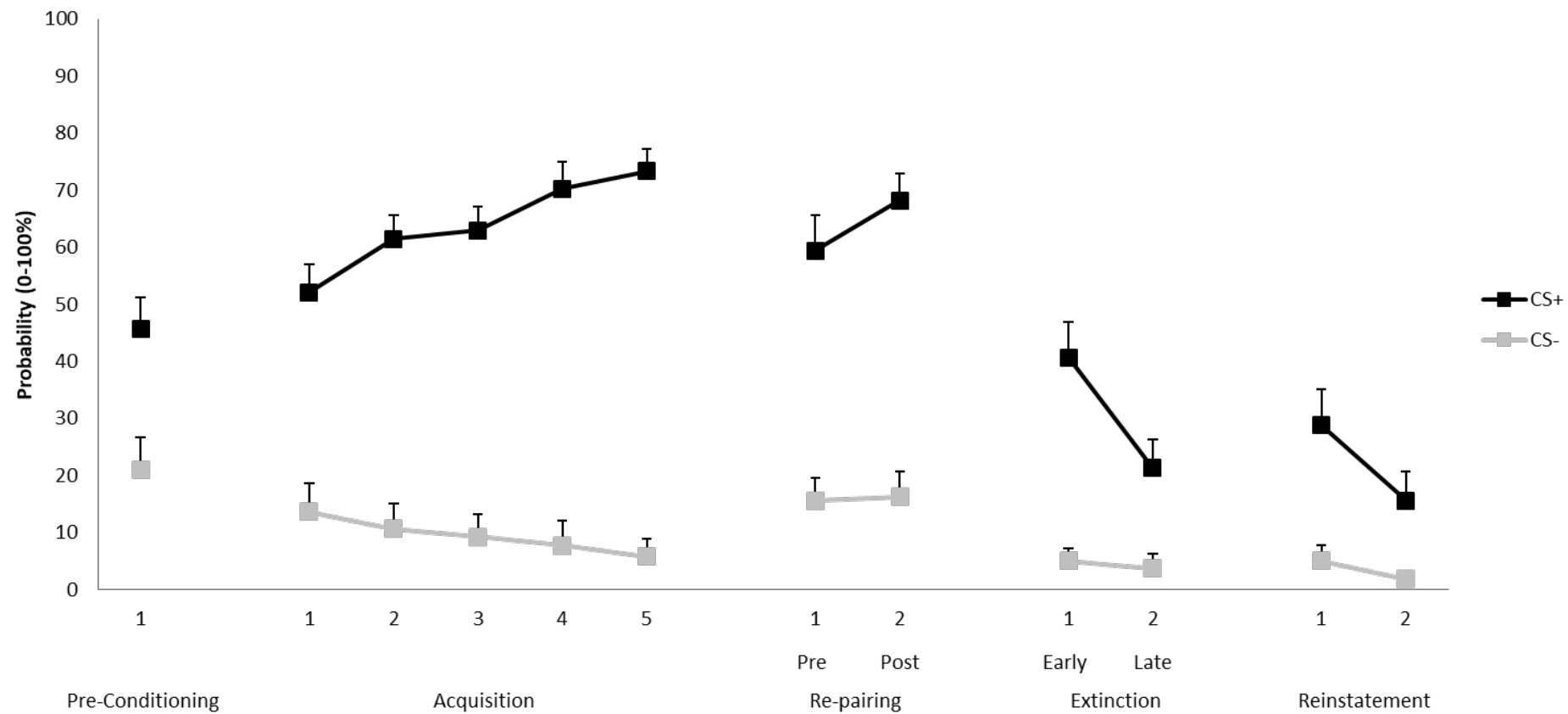

Supplement: Supplementary file 5 — Figure S1 [file 41398_2020_786_MOESM5_ESM.pdf]

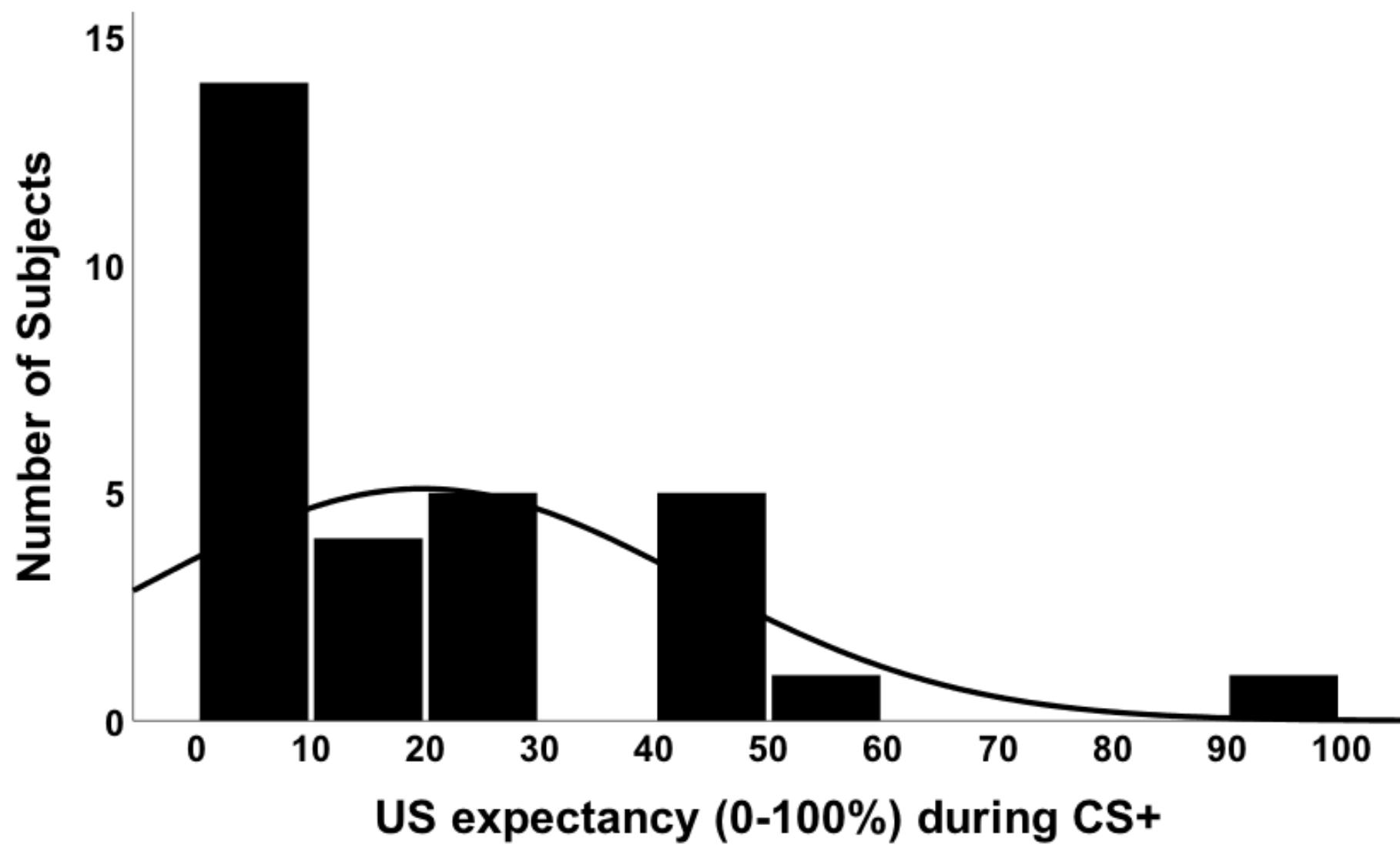

Supplement: Supplementary file 6 — Figure S2 [file 41398_2020_786_MOESM6_ESM.pdf]

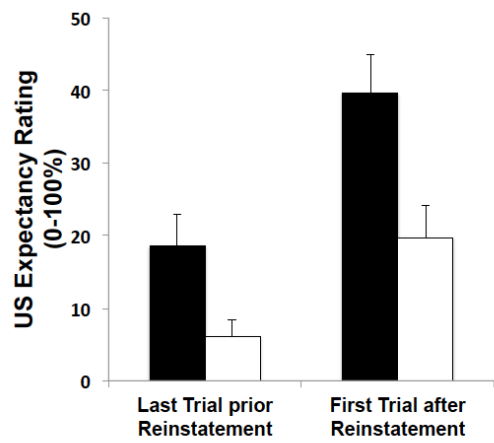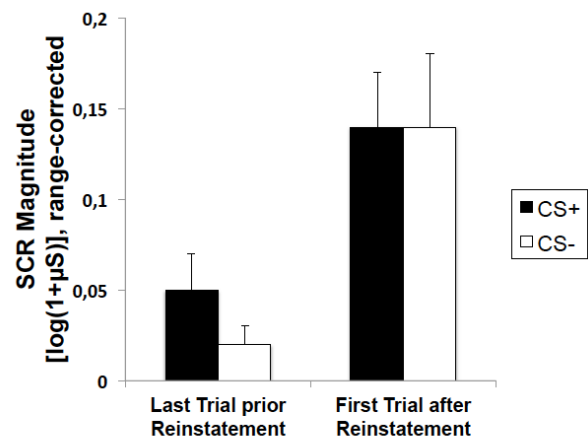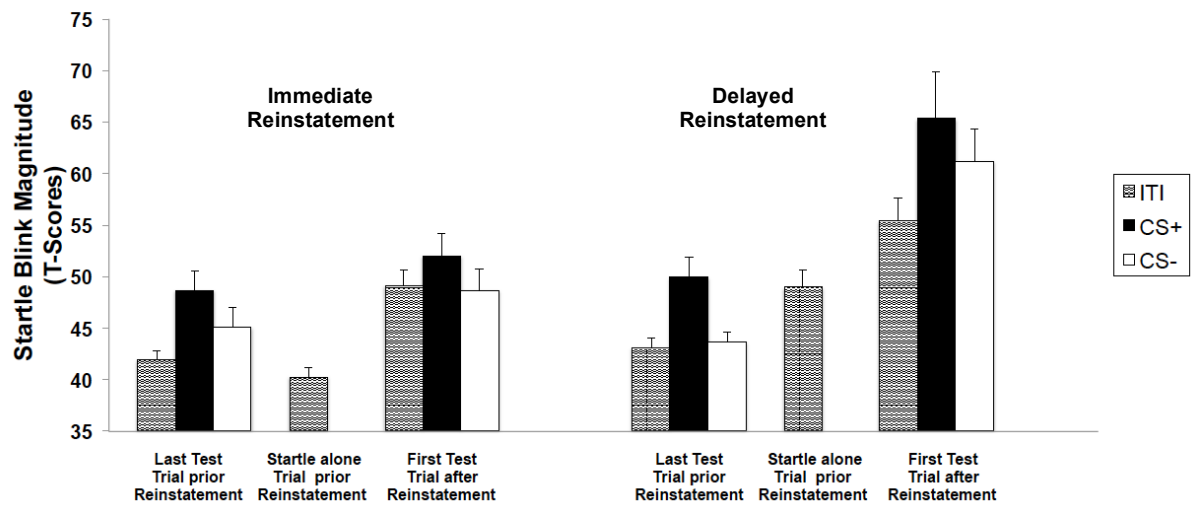

Supplement: Supplementary file 7 — Figure S3 [file 41398_2020_786_MOESM7_ESM.pdf]

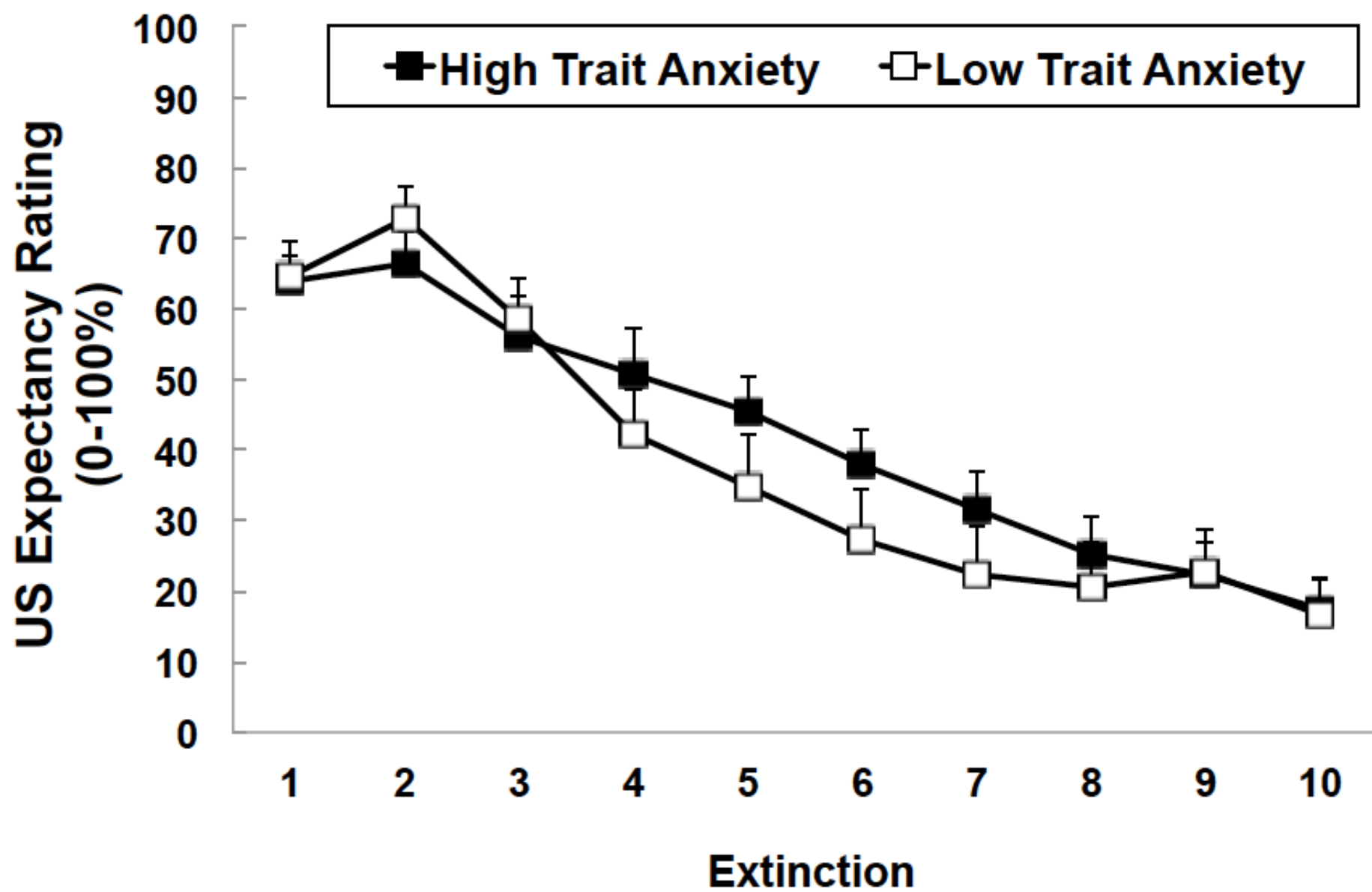

Supplement: Supplementary file 8 — Figure S4 [file 41398_2020_786_MOESM8_ESM.pdf]
